# Tinea Gladiatorum: Epidemiology, Clinical Aspects, and Management

**DOI:** 10.3390/jcm11144066

**Published:** 2022-07-14

**Authors:** Adam Zalewski, Mohamad Goldust, Jacek Cezary Szepietowski

**Affiliations:** 1 Department of Dermatology, Venereology and Allergology, Wroclaw Medical University, 50-368 Wroclaw, Poland; zalewski.adam.med@gmail.com; 2 Department of Dermatology, University Medical Center, Johannes Gutenberg University, 55122 Mainz, Germany; mgoldust@uni-mainz.de

**Keywords:** tinea gladiatorum, tinea corporis gladiatorum, *Trichophyton tonsurans*, trichophytosis, epidemiology, treatment

## Abstract

Tinea gladiatorum (TG) is a fungal skin infection that occurs among wrestlers and other contact sport athletes with a varied prevalence rate. The most common causative factor responsible as well for local outbreaks of the infection is an anthropophilic dermatophyte species—*Trichophyton tonsurans* (*T. tonsurans*). The purpose of this study was to gather current data about TG, including epidemiology, possible diagnosing methods, clinical features, treatment approaches, and potential prevention techniques. We also performed a systematic review of studies describing TG incidence. The prevalence of the disease varied from 2.4% up to 100%. That wide range of variability forces medical practitioners to update knowledge about TG and points to the fact that it still may be a diagnostic and therapeutic challenge. Spreading awareness among athletes and trainers is one of the most important preventive steps.

## 1. Introduction

Tinea gladiatorum (TG; trichophytosis gladiatorum; tinea gladiatorum) is the most widespread fungal skin infection among contact sports athletes and the second most common skin infection in this group in general, after herpes gladiatorum (HSV infection) [1,2]. Specific factors such as skin-to-skin contact during training and competition, exposure to mechanical trauma (abrasions and cuts), and often asymptomatic course of the disease may increase the risk of contamination and local outbreaks [3,4]. According to a study conducted by Iranian scientists [5], the most frequently identified pathogen causing TG was *Trichophyton tonsurans* (*T. tonsurans*) (92%) followed by *Trichophyton rubrum* (*T. rubrum*) (3.36%) and *Trichophyton mentagrophytes* (*T. mentagrophytes*) (1.89%). Kohl et al. [4], during the evaluation of association of superficial dermatophytosis and athletic activities, reported that in 84% of the studied wrestling teams in the USA during the 1998–1999 season, at least one athlete was diagnosed to be a *T. tonsurans* carrier. *T. tonsurans* is a strongly transmissible, ubiquitous anthropophilic dermatophyte fungus that originated in South-East Asia and Australia. It is a microorganism that invades keratinized tissues [6,7].

The main issue of this study was to summarize the current knowledge about the epidemiology, diagnosing, clinical features, treatment, and prevention of tinea gladiatorum.

## 2. Materials and Methods

**Figure 1:**
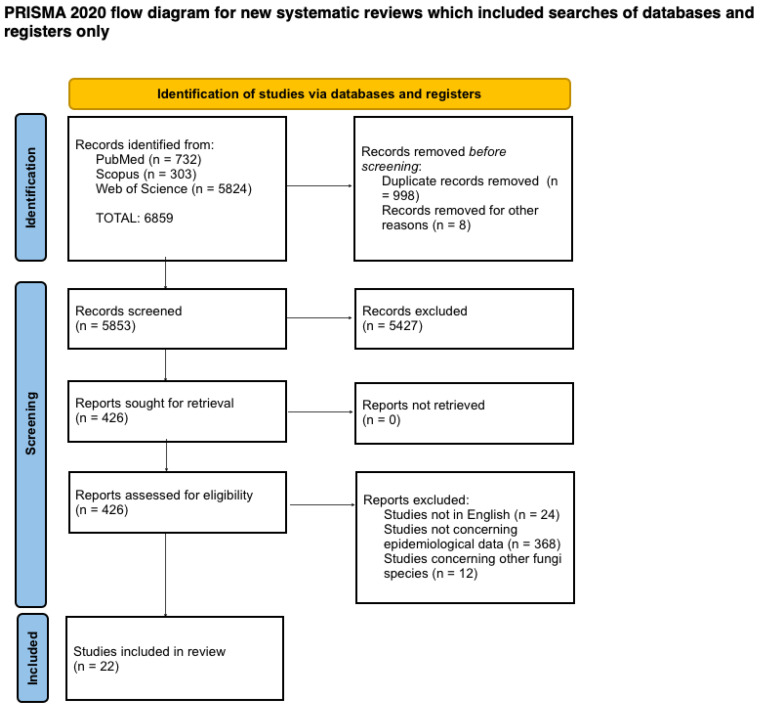
PRISMA flow diagram.

In this study, we performed a review of the literature concerning tinea gladiatorum incidence according to the PRISMA guidelines [8]. Three electronic databases (“PubMed”, “Scopus”, and “Web of Science”) were searched on 20 June 2022 for articles of any type published since 1992. The following medical subject heading (MeSH) terms were used: “tinea gladiatorum” OR “fungal infection” OR “dermatophyte” OR “dermatophytosis” OR “tinea corporis” OR “contact sports” OR “*Trichophyton tonsurans*” AND “wrestler(s)”. After the first search, we gathered 6859 articles. Later on, after removing duplicated articles, we obtained 5853 papers. After the first screening, we found 426 records; however, after a precise review of titles and abstracts and assessment of eligibility (during which all articles that were non-English or not concerning sufficient epidemiological data or concerning other fungi species were excluded immediately from the study), we extracted a total number of 22 elements which were included in our review. The PRISMA flow diagram was constructed (Figure 1). All initially searched articles were imported to Zotero 5.0, AGPL license.

## 3. Results

Of all collected articles, 22 records provided quantitative data about TG frequency among contact sports athletes. Results of our research are presented in Table 1. The analysis of the data revealed that the prevalence of dermatophytosis in wrestlers varied from 2.4% in Iran [9] up to 100% in the USA [10,11]. What was underlined by most authors was that *T. tonsurans* was undoubtedly the pathogen responsible for most cases of the infection.

## 4. Discussion

### 4.1. Pathophysiology

*T. tonsurans* is a microorganism of a filamentous fungi group, dermatophytes (subdivided into seven genera: *Trichophyton*, *Microsporum*, *Epidermophyton*, *Paraphyton*, *Lophophyton*, *Arthroderma*, and *Nannizzia*), which are keratinophilic and keratinolytic pathogens [30,31]. Based on their main host species, they are classified as anthropophilic, zoophilic, or geophilic. When involving skin infections, this type is called tinea or dermatophytosis. Many ways of transmission are possible (direct contact with infected humans, animals, or soils or indirectly via contaminated objects) [32].

Dermatophytosis, such as TG, concerns the stratum corneum in cases where the skin is occupied [3]. *T. tonsurans* as a dermatophyte species is able to adhere to host keratinized tissues and to adjust to the local environment thanks to various fungal enzymes (collagenolytic, elastolytic, and mucolytic; lipases and nucleotidases) and proteins which are regulated by specific, multiple genes. The balance between their activation and repression, depending upon the host factors (such as skin pH), provide optimum conditions for disease development [33]. Fungal heat shock proteins and transcription factors (e.g., Hfs1) are significant, especially in the first phase of the infection. Thanks to them, pathogens can adapt to the acidic pH of the hosts′ skin, and later on in the course of the infection, it may be gradually altered into alkaline. In this specific environment, keratinolytic proteases operate at maximum efficiency and ensure the continuation of the infectious process [33,34].

When considering the scalp area, *Trichophyton*, including *T. tonsurans*, also infects the hair and hair follicles, which leads to endothrix hyphae invading the hair shaft as well as internalizing into the hair cell and being converted into arthroconidia (spores). They substitute hair cells almost entirely and are visible inside the hair. Sometimes, it leads to hair breaking off at or below the mouth of the follicle, leaving the residual part inside the follicle, which gives an impression of black dots on a smooth, bald background and thus a black-dot ringworm” appearance [3,35,36,37].

Molecular tests conducted by Sidrim et al. [38] showed a potentially important role of melanin synthesis in *T. tonsurans* infection. Production of this molecule by fungi may act as an antioxidant that protects its cells from the negative impact of UV rays. It can help the pathogen to survive undamaged, e.g., in lesions that are directly exposed to the sun (on the scalp area) [38].

### 4.2. Epidemiology

Dermatophytic skin infections are one of the most frequent infections worldwide, also among contact sports athletes. Comparing data that we collected during our paper′s preparation, the range of positive mycological test results in groups of wrestlers varied significantly between different countries. The most noticeable dissimilarity was observed among athletes in the USA (results ranged from 4.3% to 100%) [10,11,12,13,15,16,18,29] and Turkey (from 4.2% to 90.6%) [20,22,23,26]. We also noted a diversity among Iranian studies (from 2.4% to 68.3%) [1,9,21,24,25,28]. There are limited data concerning the incidence of TG in other countries—only individual studies have been performed. A research involving French combat sports players showed that 37.4% of the results of mycological tests of the examined group were positive [19]. A similar study on Spanish athletes detected a fungal infection in 44.1% of analyzed individuals [17]. The percentage of positive results was relatively higher in Mexican and Swedish investigations (respectively: 57.1% [27] and 73.7% [14]). In 2015, Hiruma et al. [6] demonstrated a review of their work results concerning the epidemiology of *T. tonsurans* infection in Japan. They created a group of a total of 1000 judo wrestlers and tested them for *T. tonsurans* infection presence. The results showed that 11.5% of combat sports players were positive and 40% of that positive group presented the symptoms of tinea corporis for more than half a year. Moreover, to compare the prevalence of *T. tonsurans* infection among athletes in other disciplines, they tested 497 students in a university department of sport science—*T. tonsurans* infection was detected in combat sports players alone. Outcomes of this study indicate the need for rising patient awareness, especially among that specific group.

Bassiri-Jahromi et al. [39] in their study of superficial fungal infection in Iranian athletes demonstrated that in almost 4.3% of cases, tinea corporis was present not only among athletes but also among their family members. Thus, during wrestlers’ diagnosing and treatment, the social and domestic dimension of the contagiousness need to be taken into consideration. It concerns especially large families, herded in the crowded living conditions or facing social or financial problems, which stays in line with other authors’ observations [6,40].

Despite the fact that *T. tonsurans* is a predominant infectious factor responsible for TG cases, there are also several papers describing the role of other dermatophyte species such as *T. mentagrophytes* [41,42]. In 2000, Skorepová et al. [41] published a report informing about a case of two patients (wrestlers) with skin lesions diagnosed as TG. The probable primary source of infection was a pet rabbit owned by one of the athlete’s girlfriend, and the disease spread further by skin-to-skin contact. Symptoms of dermatophytosis were also present in four other members of the same wrestling team. *T. mentagrophytes* was diagnosed as a pathogen responsible for the infection.

It is also worth noticing that *T. rubrum* appears to be the most common causative agent of dermatophytosis [34] overall and yet it is described as the second most frequent pathogen among wrestlers with fungal infections [5]. Considering the above mentioned evidence, *T. rubrum* may be considered as a future candidate for a prevalent TG-inducing factor.

### 4.3. Risk Factors

So far, many risk factors of TG have been identified including, e.g., age, sex, skin type, hereditary factors, and hyperhidrosis [39]. The group of Shiraki et al. [43] in 2006 also described some other variables, such as a failure to wear headgear during combat, a failure to wash clothes at least once a week, and a history of scalp and neck involvement. Several of those assumptions were confirmed by the research of Turkish scientists [22]. They observed a potential role of not wearing headgear and lack of bedding laundering in local outbreak development. Training conditions are also certainly not without significance. Many athletes practice in tight, enclosed, and humid spaces. On the one hand, it may be a part of endurance training, but alongside increased sweating and risk of skin injury, it may also escalate the chance of skin-to-skin dermatophyte transmission [1,28].

The relevance of wrestling mat and wrestling hall contamination is an extensively debated factor. Some authors imply that contamination of wrestling mats with *T. tonsurans* plays a great role in dermatophytosis spread [1,24]. Constant contact between wrestlers’ feet and mats should point to the lower extremities area as the mostly affected body parts. According to a meta-analysis of TG cases conducted by Kermani et al. [5], the trunk, scalp, and face are three predominant disease locations, which would exclude the above mentioned hypothesis.

Nevertheless, colonies of *T. tonsurans* have been repeatedly isolated from sampled wrestling mats [1,24]. It is believed that training mats may be the vector of the pathogen but may not be a reservoir if the cleaning routine is proper [44].

### 4.4. Clinical Features

#### 4.4.1. Location 

**Figure 2:**
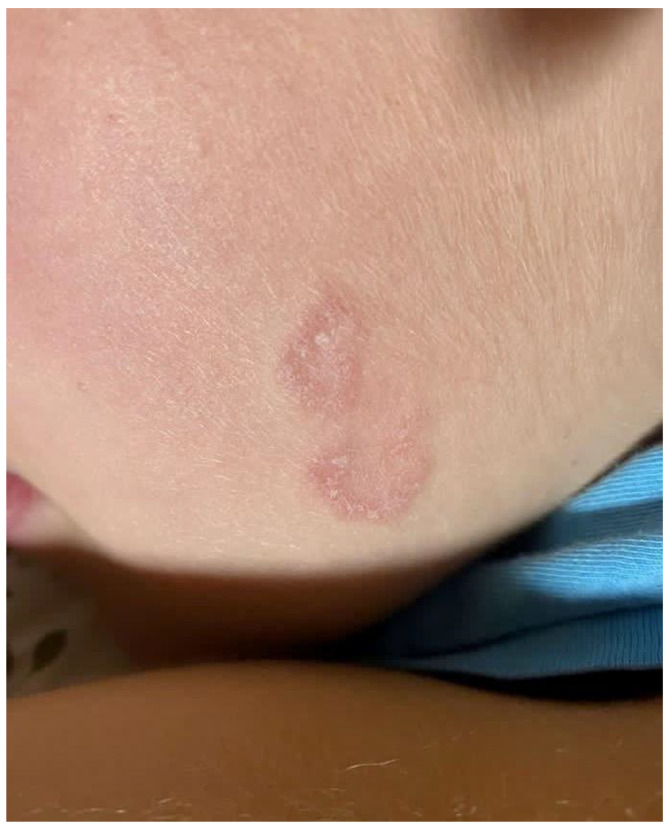
Clinical picture of TG—annular lesion with raised borders, slight scaling, and mild erythema. Courtesy of Prof. J. C. Szepietowski.

Most lesions on wrestlers are found on the surface of the trunk, head, and neck and on the upper extremities—which are points of contact between competitors—whereas the lower limbs are a rather untypical location [1,5,45]. Historically, the term tinea gladiatorum has been limited to tinea corporis in the trunk area. However, while preparing our study, we collected data describing *T. tonsurans* infections concerning other body areas of wrestlers, such as the head (*tinea capitis*), face (*tinea faciei*) (Figure 2), groins (*tinea cruris*), feet (*tinea pedis*), and nails (*tinea unguium*) [1,20,46].

#### 4.4.2. Lesion Characteristics 

Lesions caused by dermatophytes are usually clinically distinctive and can be differentiated by experienced dermatologists. Nevertheless, the clinical picture of dermatophyte infection may differ and depend on the pathogen’s species, the affected anatomic area, and the immunological response of the patient [32,35]. Taking into consideration all above mentioned evidence, disease manifestation diverges from asymptomatic carriers with no perceptible symptoms to patients presenting acute inflammatory reactions. *T. tonsurans* infection may imitate other skin conditions such as impetigo [35]. In contact sports athletes, dermatophytic lesions can also mimic abrasions resulting from, e.g., mat burns. The quantity of lesions is often underestimated, especially in cases when they occur on the surface of the back and the scalp where self-observation is rather difficult [19]. Types of lesions according to the possible site of infection are presented in Table 2.

##### Trunk

Lesions on the surface of the trunk can be described as well-defined and annular scaling papules and/or plaques with raised borders, mild erythema, and a clear center. Lesions may vary in size, usually ranging from 1 to 5 cm, but larger lesions can also occur. They can be single as well as multiple [5,39,46,47,48]. Three clinical types are distinguished: tinea circinata type, eczema marginatum type, and plaque-like type [35]. Early lesions might be difficult to differentiate from atopic dermatitis, psoriasis, early herpes gladiatorum, or acneiform papules. Additionally, sometimes they heal spontaneously, leaving the patient unaware of remaining a pathogen transmitter [35,47,48].

##### Scalp

A typical clinical picture of *T. tonsurans* infection in the scalp area can be generally characterized as a ‘black-dot’-pattern alopecia with a scaling, patchy distribution of hair loss or scalp inflammation. In some cases, lesions on the scalp are accompanied by erythematous, scaly macules or annular plaques on the face [20,40].

In this particular region, TG can be divided into three major clinical types [6]:

- Black-dot ringworm type—a result of a hair invasion (*endothrix*) [37]. In dermoscopic assessment, they can be found as a “comma-shaped” hair—a short hair with homogenous pigmentation and thickness [49,50,51]. Rarely, it is reported on other body sites such as the trunk or extremities [37,52,53];
- Seborrheic type—it can mainly be characterized by scalp scaling and the presence of crusts;
- Kerion celsi type—a rare inflammatory manifestation of tinea capitis which represents a delayed T cell-mediated hypersensitivity reaction. It is an intense immune response to the infection caused by dermatophytes which results in limited, infiltrated, suppurative, and tender lesions. Sometimes, cervical lymphadenopathy and id reactions are also reported. If not treated, it may cause scarring alopecia. Kerion celsi needs to be differentiated from cellulitis, seborrheic dermatitis, and carbuncle. Most cases concern the pediatric population. Sporadically, lesions may also arise on other parts of the body, e.g., eyebrows or vulva [54,55,56,57,58,59].

According to an Irish study conducted on the pediatric population, *T. tonsurans* infection accounted for 75.8% of tinea capitis cases [60]. In adults, the sebum consists of, i.a., triglycerides and short-chain fatty acids, which play a great role in preventing infection because of their fungistatic features [30].

### 4.5. Quality of Life

Like many other skin conditions, dermatophytosis also has an undeniable impact on patients’ quality of life. According to the study performed in 2013 to assess the global burden of disease, superficial fungal infection is the fourth most frequent cause of illness globally [61,62]. Dermatophytosis significantly impacts patients’ quality of life by inducing psychosocial consequences such as embarrassment, low self-esteem, anxiety, and depression, the main explanation of which is attributed to pruritus and aesthetic matters [62,63]. In 2019, the group of Narang et al. [62] performed a study investigating the quality of life of patients with dermatophytosis. A total of 196 subjects participated in this survey. The mean total Dermatology Life Quality Index (DLQI) score reached 13.41 ± 7.56 points (range 0–30 points). Items described to be mostly influenced were “symptoms and feelings”, “daily activities”, “leisure”, and “personal relationships”. Both age and body surface area had a significant impact on patients’ quality of life (QoL) (*p* ≤ 0.05). Mushtaq et al. [64] also proved that the DLQI score may be influenced by the severity of the disease. 

### 4.6. Diagnosis

In most cases, the diagnosis is made on the basis of the clinical picture. In some wrestling teams, not only were the physicians and the wrestlers themselves involved in the diagnostic process, but the coaches were also included. What is more, some of them admitted to deciding if the athlete is permitted to continue with training without a doctor′s consultation [65].

The obligatory first step of dermatophytosis diagnosis should be a precise clinical examination conducted by a physician, preceded by taking an extensive patient history (possible vectors; contact with infected persons and animals; hobbies; socioeconomic situation). Typical morphological figures as well as the pattern of lesion distribution may be helpful in distinguishing the disease. 

Another basic mycological test that is widely performed is a microscopic inspection of material, such as skin scrapings, hair, or nails in KOH mounts (*potassium hydroxide*). A 10–20% KOH solution acts as a macerating agent and allows the diagnostician to observe dermatophyte hyphae [66]. Sometimes also, specific stains (e.g., Parkerink, Chicago blue) may be used in specimens to mark fungal elements [67]. Histological examination performed with the use of periodic acid schiff (PAS) stain can reveal signs of tinea [68].

There are also many methods that support medical practitioners and let them approach the specific diagnosis. For instance, inspection with Wood′s light allows to screen for Microsporum canis infection [32], and dermatoscopy enables to differentiate tinea capitis from other dermatoses [51]. The latter is also used in cases of onychomycosis (distinguishing post-traumatic damages from fungal infection [69,70,71]) or to exclude tinea nigra in palmar or plantar lesions [72]. Other more advanced techniques of fungi detection are optical coherence tomography and confocal laser scanning. The most important advantages of their use are non-invasiveness and immediate real-time test results. On the other hand, particular technical skills are needed to understand and properly interpret those outcomes. The availability of these methods is, however, rather low [73,74].

**Figure 3:**
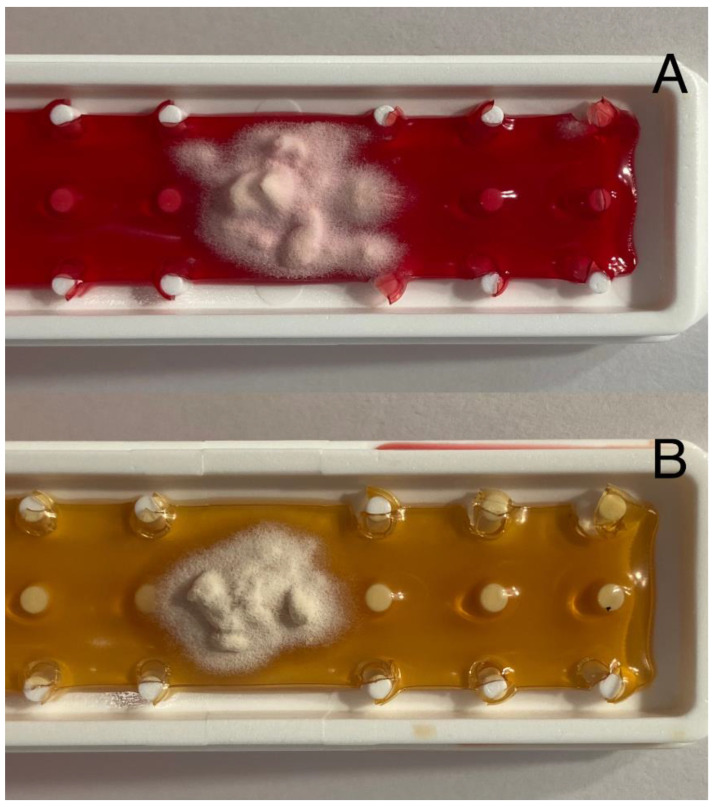
Pictures of *Trichophyton tonsurans* culture: (**A**) Fungiset agar growth medium with actidione (cycloheximide) and phenol red; (**B**) Sabouraud dextrose agar growth medium with chloramphenicol. Courtesy of Prof. J. C. Szepietowski.

**Figure 4:**
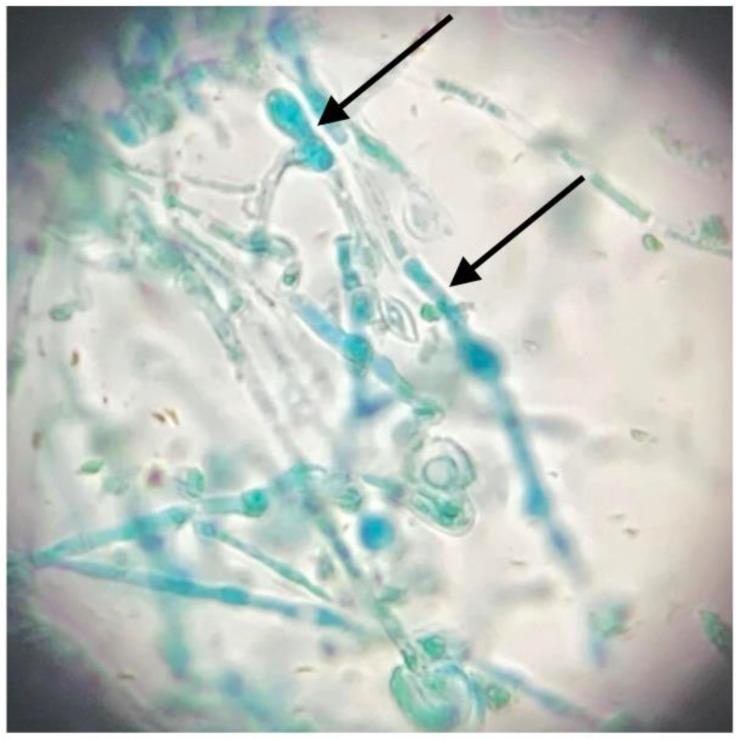
Microscopic picture of microculture: numerous microconidia, including club-shaped ones (pointed by black arrows). Courtesy of Prof. J. C. Szepietowski.

Tracing epidemics is one of the most important tasks and challenges of public health and modern epidemiology. Moreover, helping to determine the source of infection may be crucial in restricting the spread of the pathogen [75]. A classical procedure of dermatophyte identification is growing a culture on a specific medium such as Sabouraud dextrose agar. One of the agar plates should also contain cycloheximide to prevent bacteria and non-dermatophyte mold contamination [76] (Figure 3 and Figure 4).

Molecular biology methods are presumably the most rapidly developing branches of laboratory diagnostics and are widely used for identifying particular microorganisms as well as dermatophytes. To obtain results confirming the presence of specific species of the pathogen, different parts of fungal DNA are used [77]. Genetic methods involve the polymerase chain reaction (PCR) fingerprinting technique with single arbitrary primers, the analysis of restriction fragment length polymorphism (RFLP) of mitochondrial DNA, or comparison of ribosomal DNA sequences [78]. An analysis of *T. tonsurans* strain’s molecular marker polymorphisms (e.g., NTS (*non-transcripted spacer*) regions of ribosomal RNA gene (rDNA)) performed by American researchers presented significant strain-specific genetic variations [79]. Different strains may present dissimilarities in pathogenicity which exert an impact on the minimum inhibitory concentration (MIC) of antimycotics [75]. Furthermore, divergence in genes and recognizing them brings information about virulence of the fungi and specific infection patterns [38].

Identification of dermatophytes can also be performed based on mass spectrometry (MS) methods such as matrix assisted laser desorption/ionization time-of-flight (MALDI-TOF) analysis. In this assay, fungal material is evaporated by a laser beam and ionized particles are analyzed by mass spectrometry according to their time of flight [32,80]. Erhard et al. [81] in 2007 proved that the results of species identification by MALDI-TOF mass spectrometry are comparable with those obtained by using molecular methods (fungal DNA sequencing). Nevertheless, MALDI-TOF MS has also some limitations such as reliability on the quality of the reference spectra library [80].

The variety of methods for detection and identification of dermatophyte species and strains is constantly expanding. However, that process would not be possible without comparison to most efficient and reliable classical mycological techniques. It should also be emphasized that mycological tests are essential for proper tinea management.

### 4.7. Treatment

Management of dermatophytic infections consists of topical antifungals individually when disease is limited or oral therapeutics individually or in combination with topical agents in disseminated or resistant cases.

#### 4.7.1. Topical

Numerous topical antifungal formulations that are effective in tinea gladiatorum treatment have been reported. Most topical antifungals are prescribed once or twice daily for 2–4 weeks [82]. In 2013, Rotta et al. [83] created a meta-analysis of the efficacy of antifungal treatment of 14 different topical antifungals. Their study included 65 randomized controlled trials, where pharmaceuticals were compared with one another and with placebo. No statistically significant differences between the antifungals were reported based on the result of mycological cure at the end of treatment. To achieve a long-lasting recovery, butenafine and terbinafine were found to be superior to clotrimazole, oxiconazole, and sertaconazole; terbinafine to ciclopirox; and naftifine to oxiconazole. A similar result to terbinafine antifungal activity was also shown by luliconazole [84]. A Cochrane review concerning topical antifungals demonstrated that terbinafine and naftifine in monotherapy are effective in dermatophytic infections and adverse effects occur rarely. Other antifungals such as azoles were reported to be effective ways of management as well [82]. Not only is the drug part of the preparation important but also the medium. Research conducted on lipid-based amphotericin B gel or microemulsion has shown promising results [85,86]. A novel film-forming terbinafine solution presented fungicidal effects that lasted almost 2 weeks after only a single application [87].

#### 4.7.2. Systemic

Although topical agents are a first-line treatment, in some cases, this kind of therapy may be unsuccessful, and lesions can also be so vast that local pharmaceuticals appear to be helpless. Some locations, such as the back of the trunk, are also difficult to self-reach [88]. In those patients, oral antifungals need to be prescribed. It also concerns patients with affected surface of the scalp (tinea capitis). 

Itraconazole and terbinafine are the most commonly chosen pharmaceuticals. Daily doses are 100–200 mg and 250 mg per day, respectively, used for 1–2 weeks [76]. 

Other alternatives are fluconazole (150–200 mg once weekly) and griseofulvin (500–1000 mg daily). The latter is no longer used in some European countries [89]. Those options, however, demand long-term use (2–4 weeks) [76].

In 2020, the group of Kermani et al. [90] performed a study to assess the in vitro activity of the most common antifungals used against *T. tonsurans* obtained from wrestlers with different types of dermatophytosis (tinea corporis, tinea capitis, tinea manuum, tinea faciei, and ținea cruris). They tested eight most commonly prescribed antifungal agents and concluded that tolnaftate and itraconazole had the most potent antifungal activity (concerning *T. tonsurans* infection). Fluconazole seemed to present the weakest effect [90].

However, what should definitely be alarming is that with each year, there is an increasing number of reports informing about dermatophyte resistance and tolerance of antifungals [34,91]. Some mechanisms responsible for that which were lately outlined are, e.g., drug efflux, drug degeneration, overexpression of chaperones, target mutation, and biofilm formation. Factors increasing the possibility of drug resistance are long-term therapy and discontinuation of treatment against a doctor′s recommendations [34]. Even more disturbing is the fact that also a cross-resistance was described, showing decreased sensitivity to allylamines and azoles. The group of Singh et al. [92] reported a considerably high terbinafine resistance rate of 32% among 63 *T. interdigitale* isolates and cross-resistance to itraconazole, fluconazole, sertaconazole, voriconazole, and griseofulvin.

### 4.8. Prevention

Diagnosed infection leads to the loss of competition and practice time which may influence athletes’ both physical and mental condition. It can also affect the whole team [29,93]. There are methods and measures that are undertaken to prevent disease transmission and to limit local outbreaks. Those strategies, including wrestling mat sanitization (benzalkonium chloride might be particularly effective [27]), washing by wrestlers immediately after the practice, pretreatment with barrier foam before an encounter, and mandatory skin inspection protocols preceding each competition, might spectacularly decrease the number of TG cases [29,46,93,94]. In 2009, American scientists from Toledo published the results of their prospective longitudinal study that was conducted among a group of 373 wrestlers between 1997 and 2007 and concerned the evaluation of the use of prophylactic oral fluconazole in reducing the frequency of tinea gladiatorum. Athletes received 100 mg fluconazole in tablets for 3 days prior to the wrestling season opening. That 3-day dosage was repeated in week 6. Over the course of the study, the incidence rate of TG decreased from 67.4% to 3.5%. No adverse effects of the intervention were observed [95]. Another possibility described in the literature is itraconazole pulse therapy. The group of Hanzen et al. [15] created a survey in which they prescribed wrestling team members oral itraconazole, 200 mg, twice a day for 1 day every 2 weeks. No signs of active tinea were found among examined athletes during the follow-up period (half of a wrestling season). To prevent infection, talcum powders may also be used, often combined with antifungals such as clotrimazole or terbinafine. They absorb excessive amounts of sweat, reduce the moisture of the skin, and simultaneously act against dermatophytes [76]. Moreover, authors have underlined the obvious role of athletes’ and trainers′ education in outbreak prevention [45,93].

## 5. Conclusions

Tinea gladiatorum was and continues to be one of the most frequent infectious skin diseases among wrestlers. Although the infection does not cause serious life-threatening consequences, it may influence individual and team sport goals. It also impacts patient quality of life. Over the course of years, many risk factors have been found, and what is more, contact sports athletes, trainers, and medical practitioners taking care of them has shown that awareness of fungal skin infections definitely has increased. A precise physical examination followed by proper mycological tests forms the basis of tinea gladiatorum diagnosis. Both topical and systemic antifungals’ formulations allow dermatologists to choose the best line of treatment and to escalate it if required. Preventive steps need to be undertaken to limit the amount of new cases and restrain local outbreaks. Not only athletes themselves but also their family members must be taken into account as potential pathogen carriers. 

## Figures and Tables

**Table 1:** Tinea gladiatorum (TG) frequency among contact sports athletes.

Author	Country	Publication Year	Positive Mycological Results in Wrestlers/Total Number of Samples (% Value)	*T. tonsurans*-Positive Samples
Cohen et al. [12]	USA	1992	8/22 (36.4%)	5
Stiller et al. [10]	USA	1992	5/5 (100%)	5
Werninghaus et al. [11]	USA	1993	4/4 (100%)	No data
Beller et al. [13]	USA	1994	21/28 (75%)	10
Hradil et al. [14]	Sweden	1995	14/19 (73.7%)	14
Hazen et al. [15]	USA	1997	10/37 (27%)	No data
Kohl et al. [16]	USA	1999	22/63 (34.9%)	22
Pique et al. [17]	Spain	1999	45/102 (44.1%)	No data
Adams et al. [18]	USA	2000	7/29 (24.1%)	No data
Poisson et al. [19]	France	2005	49/131 (37.4%)	48
Ergin et al. [20]	Turkey	2006	29/32 (90.6%)	20
Hedayati et al. [1]	Iran	2007	65/324 (20%)	65
Bassiri-Jahromi et al. [21]	Iran	2008	612/893 (68.5%)	566
Ilkit et al. [22]	Turkey	2010	14/29 (48.3%)	14
Ilkit et al. [23]	Turkey	2011	17/194 (8.7 %)	11
Aghamirian et al. [24]	Iran	2011	52/270 (19.3%)	43
Habibipour et al. [9]	Iran	2012	44/1800 (2.4%)	44
Ahmadinejad et al. [25]	Iran	2013	17/454 (3.7%)	11
Dogen et al. [26]	Turkey	2013	6/143 (4.2%)	2
Bonifaz et al. [27]	Mexico	2020	4/7 (57.1%)	4
Kermani et al. [28]	Iran	2020	278/4240 (6.5%)	192
Berg et al. [29]	USA	2021	22/510 (4.3%)	No data

**Table 2:** Types of lesions according to the possible site of infection.

Possible Site of Infection	Type of Lesions
Trunk (*tinea corporis*)	- *tinea circinata* type - *eczema marginatum* type - plaque-like type
Scalp (*tinea capitis*)	- black-dot ringworm type - seborrheic type - kerion celsi type
Face (*tinea faciei*)	no specific types
Groins (*tinea cruris*)	no specific types
Feet (*tinea pedis*)	no specific types
Nails (*tinea unguium*)	no specific types

## Data Availability

All data are included in the manuscript.
